# Emerging Gene Therapies for Alzheimer’s and Parkinson’s Diseases: An Overview of Clinical Trials and Promising Candidates

**DOI:** 10.7759/cureus.67037

**Published:** 2024-08-16

**Authors:** Will S Roberts, Shawn Price, Michael Wu, Mayur S Parmar

**Affiliations:** 1 Osteopathic Medicine, Dr. Kiran C. Patel College of Osteopathic Medicine Nova Southeastern University, Clearwater, USA; 2 Foundational Sciences, Dr. Kiran C. Patel College of Osteopathic Medicine Nova Southeastern University, Clearwater, USA

**Keywords:** gene therapeutics, challenges and opportunities, clinical trials, neurodegenerative diseases, adeno-associated virus (aav), parkinson' s disease, alzheimer's disease, gene therapy

## Abstract

Gene therapy as a disease-modifying therapeutic approach for neurodegenerative diseases, such as Alzheimer’s disease (AD) and Parkinson’s disease (PD), is a promising avenue. Promising results in the preclinical studies involving rodents and nonhuman primates utilizing gene therapy have led to multiple clinical trials evaluating various genes of interest for AD and PD. In AD, clinical trials are assessing gene therapy involving brain-derived neurotrophic factor (BDNF) and other targets such as apolipoprotein E2 (APOE2) and human telomerase reverse transcriptase (hTERT). In PD, clinical trials are evaluating gene therapy delivering neurotrophic factors, such as glial cell line-derived neurotrophic factor (GDNF). Additionally, gene therapy delivering enzymes aromatic L-amino acid decarboxylase (AADC) and glutamic acid decarboxylase (GAD) are also being evaluated for PD. All these trials primarily utilized adeno-associated virus (AAV) to deliver the above transgene of interest. This review summarizes the current clinical trials involving gene therapy for AD and PD. It also discusses the challenges and opportunities associated with the gene therapy approach in AD and PD and ongoing developments related to increasing the safety and efficacy of the gene therapy for long-term outcomes, which include evaluation of various serotypes and administration routes. This comprehensive review emphasizes translating preclinical findings into clinical trials, further directions, and the potential for this promising therapeutic approach to alleviate neurodegenerative disease.

## Introduction and background

Neurodegenerative diseases, such as Alzheimer's disease (AD) and Parkinson's disease (PD), represent a growing public health challenge globally. These diseases have a devastating impact on individuals affected and their caretakers. AD, the most common form of dementia, affects millions worldwide and is characterized by progressive cognitive decline, memory loss, and behavioral changes. Factors contributing to the disease pathophysiology include amyloid-beta (Aβ) accumulation and tau hyperphosphorylation, resulting in the formation of neurofibrillary tangles (NFTs), in addition to neuroinflammation, immune response, and mitochondrial and autophagy dysfunction [1-4]. AD affects an estimated 6.9 million individuals aged 65 and older in the United States (US), projected to double by 2060 (13.8 million) unless effective interventions emerge [5]. PD, the second most common neurodegenerative disease, is marked by motor symptoms like bradykinesia, tremors, and rigidity. These symptoms arise due to the degeneration of dopamine-producing neurons in the substantia nigra pars compact [6-9]. PD impacts over one million individuals in the US and more than six million worldwide [10]. Both diseases have a high prevalence rate as well as diverse underlying genetic and molecular mechanisms.

The current treatments for both diseases primarily focus on symptom management and offer limited efficacy in slowing or halting disease progression [3,10]. However, recently approved disease-modifying therapies for AD, such as Aduhelm® (aducanumab-avwa), Leqembi® (lecanemab-irmb), and Kisunla® (donanemab-azbt), target Aβ pathology through distinct mechanisms and represent significant advancements in treatment.* *Despite these developments, the absence of preventative or curative therapies underscores the urgent need for innovative therapeutic approaches. With the recently approved disease-modifying monoclonal IgG1 antibodies, there are risks associated with amyloid-related imaging abnormalities (ARIA) and concerns related to long-term effects. Consequently, there is growing research interest in developing disease-modifying therapeutics, which include utilizing a gene therapy approach.

Gene therapy involves replacing a defective gene with a healthy copy, inactivating a malfunctioning gene, or introducing a new gene to alleviate the disease [3,10-13]. Gene therapy strategies involve using viral and nonviral carriers, called vectors, as a delivery tool to address disease-specific cellular dysfunction in the diseased condition. Extensive ongoing research is evaluating various vectors, including adeno-associated virus (AAV) and lentivirus, and their structural modifications. It also evaluates administration routes to deliver the transgene of interest tailored to the disease and its underlying genetic mechanisms.

AAV vectors have emerged as a leading platform for gene delivery. This is AAV's safety profile, ability to transduce various cell types, and efficient transport across the blood-brain barrier [10,13-15]. Recent advancements in vector engineering have improved the potential of gene therapy for neurodegenerative diseases [2,14,16,17]. The possibilities of gene therapy for neurological disorders have been expanded by engineering specific serotypes of vectors focused on improving targeting specificity, reducing immune responses, and enhancing gene expression efficiency, along with utilizing different delivery methods like intravenous(IV), intrathecal (IT), and magnetic resonance imaging (MRI)-guided stereotactic injections [10-13,15,17]. Most treatments undergoing clinical trials employ the adenovirus for gene delivery due mainly to its adept ability to target neurons and desired specific brain cell populations and its exceptional safety profile. Lentivirus, another vector, has also been used for various trials as it has been shown to carry a larger genetic load than adenovirus. Notably, gene therapy using AAV has been successful in treating a variety of disorders. This includes hemophilia A and lipoprotein lipase deficiency, as well as several neurological conditions such as Duchenne muscular dystrophy (DMD), spinal muscular atrophy (SMA), aromatic L-amino acid decarboxylase (AADC) deficiency, and Leber congenital amaurosis (LCA). Gene editing technologies like CRISPR-Cas9 also offer the potential for even more precise and targeted therapies [18,19]. CRISPR-Cas9 holds promise for addressing genetic disorders at their root cause by directly correcting disease-causing mutations in the genome [19]. While CRISPR-Cas9 holds promise for gene editing, further research is needed to ensure its safe and effective use in clinical settings.

This review article focuses on the emerging landscape of gene therapy clinical trials for AD and PD. The review highlights key gene targets, delivery strategies, and early clinical results for both diseases. By summarizing the translation of preclinical findings into clinical trials, this comprehensive review aims to provide an overview of the current state of the gene therapy approach for these debilitating neurological conditions.

## Review

**Figure 1:**
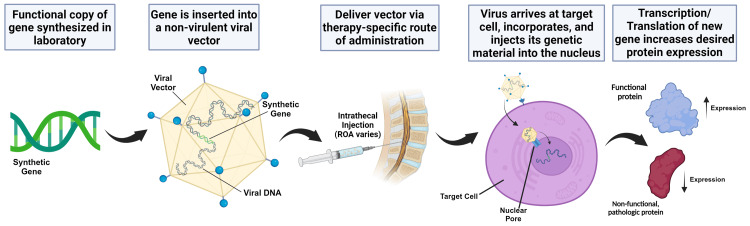
Schematic of gene therapy using a viral vector illustrating the process of gene therapy delivery and its potential impact on targeting cells (A) Synthetic gene production: a functional copy of the desired transgene is synthesized. B) Viral vector incorporation: the synthetic gene is inserted into a nonvirulent viral vector, such as adeno-associated virus (AAV). (C) Therapy-specific delivery: the viral vector carrying the therapeutic gene is administered to the patient through a specific route of administration tailored to the targeted cells. In this example, the intrathecal injection is highlighted, but the route of administration may vary. (D) Target cell infection: the engineered virus reaches the target cell and injects its genetic material, including the therapeutic gene, into its nucleus. (E) Transcription and translation: the cell's machinery transcribes the therapeutic gene into mRNA, which is then translated into a functional protein. This increased expression of the desired protein aims to correct the underlying genetic defect or disease condition. The therapeutic approach aims to restore normal protein function and alleviate the disease symptoms by introducing a functional gene copy. The image was created using BioRender.com

The Clinicaltrials.gov website was analyzed to identify all active and completed gene therapy trials for neurodegenerative diseases, specifically AD and PD (Table 1). Figure 1 highlights the mechanism of AAV infection and subsequent transgene expression.

**Table 1:** Table summarizes results from completed clinical trials and key endpoints of ongoing trials for Alzheimer's and Parkinson's diseases APOE2: Apolipoprotein E2; BDNF: brain-derived neurotrophic factor; hTERT: human telomerase reverse transcriptase; GDNF: glial cell line-derived neurotrophic factor; AADC: aromatic L-amino acid decarboxylase; GAD: glutamic acid decarboxylase; AAV: adeno-associated virus; CNS: central nervous system; CSF: cerebrospinal fluid; MCI: mild cognitive impairment; AD: Alzheimer's disease; MRI: magnetic resonance imaging; PD: Parkinson's disease: STN: subthalamic nucleus; MDS-UPDRS: Movement Disorder Society-Unified Parkinson's Disease Rating Scale; LEDD: levodopa equivalent daily dose; UDysRS: unified dyskinesia rating scale

Clinical trial identifier	Disease target	AAV serotype	Transgene	Phase	Patient enrollment	Route of administration	Key findings (as of June 2024)
NCT03634007 (LX1001)	Alzheimer's (APOE)	AAVrh10	APOE2	1 and 2	15	Intrathecal	Press release related to NCT03634007: In CSF, APOE2 protein was detected in all four participants of the cohort after three months. Following a year, two participants achieved a state where their total tau and phosphorylated tau levels had reduced from the initial baseline. There were no reports of any serious adverse events. Overall, the tolerability profile was favorable. Safety, tolerability, biomarker changes. Initial results show positive APOE2 expression and biomarker reduction
NCT05400330 (Long-term follow-up of participants from NCT03634007)	Alzheimer's (APOE)	AAVrh10	APOE2	-	15	Refer NCT03634007	Evaluation of the safety and tolerability (adverse and serious adverse events) following post-administration of LX1001 at 260 weeks
NCT05040217	Alzheimer's (BDNF)	AAV2	BDNF	1	12	Entorhinal cortex	Ongoing study in MCI and AD patients. Measurement at 24 months: Assess safety and tolerability. Changes in memory function (memory tested on Ray Auditory Verbal Learning Task and Benson Complex Figure Draw and Memory). Changes in brain imaging and biomarkers (amyloid, tau, neurofilament)
NCT04133454	Alzheimer's (hTERT)	AAV	hTERT	1	5	Intravenous and Intrathecal	Study evaluated: Safety, tolerability, hTERT expression, telomerase activity, telomere length, CNS changes, and neurocognitive function. Study completed: results not yet public
NCT01621581	Parkinson's (GDNF)	AAV2	GDNF	1	25	MRI-guided intraputaminal infusion	Reported findings [20]: Treatment was tolerated, and no clinical or radiographic toxicity was noted. Serious adverse events occurred, but they were not attributed to the AAV2-GDNF and were resolved. Average putaminal coverage of AAV2-GDNF was approximately 26% of the putaminal volume. Post-infusion increased [^18^F]-FDOPA uptake in 10 of 13 and 12 of 13 patients at 6 and 18 months, respectively. During the study, no significant changes were made to PD medications or levodopa-equivalent doses (LEDs). Two patients reduced their daily LED, however, most patients (eight in total) increased their daily LEDs, which is expected with normal PD progression. The noted changes in the specific participants could be associated with a placebo effect and/or close medical monitoring. Over the study period, UPDRS assessment scores generally remained stable. Between the different dose cohorts, no clinical or statistically significant changes in UPDRS scores were observed
NCT04167540	Parkinson's (GDNF)	AAV2	GDNF	1	11	MRI-guided intraputaminal infusion (Bilateral)	Reported findings [21]: An 18-month post-treatment, the mean putaminal coverage was 63%. Most of the treatment-emergent adverse events in all the participants were transient and perioperative. Reported serious adverse events were not related to AAV2-GDNF. Mild Cohort exhibited stable scores in MDS-UPDRS, motor diary, unified dyskinesia rating scale (UDysRS), and levodopa equivalent daily dose (LEDD). A heterozygous tyrosine hydroxylase mutation of unknown significance was noted in the one mild cohort participant. Moderate cohort demonstrated improvements from baseline at 18 months post-treatment in mean MDS-UPDRS Part III OFF scores, motor diary OFF time, UDysRS, and LEDD. Note: To confirm the findings, a phase 2 randomized controlled study is planned
NCT01973543 (VY-AADC01)	Parkinson's (AADC)	AAV2	AADC	1	15	MRI-guided intraputaminal infusion	Reported findings [22]: In subjects with PD, fluctuating responses to levodopa-dose-dependent putaminal coverage and AADC activity were observed. Less use of antiparkinsonian medication at six months. At 12 months, dose-dependent improvements in clinical outcomes were noted, which included increases in patient-reported ON-time without troublesome dyskinesia and improved quality of life. Few adverse effects were reported in these clinical trials, attributed to the surgical procedure, not the gene transduction. Reported Findings [23]: A substudy showed VY-AADC01 improved motor responses to intravenous levodopa under controlled conditions. Reported Findings [24] : At 36-month safety and clinical outcomes from the earlier trial. No serious adverse effects. Reduced medication requirements in the two highest dose cohorts at 36 months. Stable or improved motor functions (PD diary, Unified Parkinson’s Disease Rating Scale III “off”-medication and “on”-medication scores) compared with baseline at 12, 24, and 36 months following VY-AADC01 administration across cohorts. Both clinical global impression of improvement and patient global impression of improvement showed positive results. Quality of life was stable and improved compared with baseline at 12, 24, and 36 months following VY-AADC01 administration across cohorts
NCT03065192 (VY-AADC01)	Parkinson's (AADC)	AAV2	AADC	1	16	MRI-guided convective infusion using a posterior trajectory into the putamen	Reported findings [25]: Evaluated VY-AADC01 in advanced PD. Total putaminal coverage exceeded the 50% goal with a mean of 76% coverage of the post-commissural putamen. Modification to the surgical procedure reduced infusion durations and overall procedure times. As compared to the PD-1101 trial, increased AADC enzymatic activity was noted in PD-1102. VY-AADC01 post-infusion at six and 12 months, daily antiparkinsonian medication requirements were reduced. Two patients reported mild intraoperative intracerebral hemorrhage out of a total of eight who underwent AY-AADC01 administration. Postoperatively, one patient was asymptomatic, and another experienced a temporary visual disturbance. Note: The surgical and infusion techniques assessed in NCT03065192 have guided the development of the NCT03562494 (RESTORE-1) trial
NCT03562494 (RESTORE-1) (VY-AADC02)	Parkinson's (AADC)	AAV2	AADC	2	14 (actual)	Intraputameninal	Study was initiated in moderate to advanced PD patients with motor fluctuations [26]. The primary outcome was to assess the number of participants with treatment-emergent adverse events as a measurement of the primary outcome. Note: T2 MRI abnormalities were observed, and the study is placed on clinical hold. Sponsor partnership was terminated in 2021 (70)
NCT03733496 (Long-term extension study of NCT01973543 and NCT03065192)	Parkinson's (AADC)	AAV2	AADC	NA	14	Refer NCT01973543 and NCT03065192	Observational long-term extension study to assess the long-term safety for patients of prior VY-AADC01 studies: Study will measure: Long-term safety of VY-AADC01, as determined by the occurrence of AEs and SAEs, continued expression of AADC that will be measured by [^18^F]-FDOPA PET, Change from baseline in PD medications (quantified as LED), changes from baseline in motor function (as assessed by the UPDRS, in both “Off” and “On” medication states), and changes from baseline based on the PD diary (including "OFF" and "ON" times). The time frame for the evaluation is up to 8 years after the VY-ADDC01 administration
NCT00195143	Parkinson's (GAD)	AAV2	GAD	1	12	Unilateral STN administration	Reported findings [27]: Safe and well tolerated by patients with advanced PD. Improvements in UPDRS motor scores (3 months after gene therapy and persisted up to 12 months) and reduction in thalamic metabolism, as noted with PET scans (treatment hemisphere), were observed. A correlation was noted between clinical motor scores and brain metabolism in the supplementary motor area. No gene therapy-associated adverse events
NCT00643890	Parkinson's (GAD)	AAV2	GAD	2	45	STN administration with sham surgery (Bilateral)	Reported findings [28,29]: Improvements in UPDRS motor scores compared to the sham groups continued at 12 months, and changes in brain metabolism were observed. A significant decline in levodopa-induced dyskinesia at 12 months and brain metabolism in the treatment group. A correlation between baseline prefrontal cortex metabolism and clinical outcomes (changes in motor UPDRS scores) was observed. Note: The study was terminated due to financial reasons
NCT05603312	Parkinson's (GAD)	AAV (Not specified)	GAD	1 and 2	14	STN administration with sham surgery (Bilateral)	Ongoing study in PD patients: Assess safety and efficacy. Mean change from baseline to weeks 12 and 26 for the AAV-GAD groups compared to the sham group in MDS-UPDRS Part 3 score in the "off" state
NCT05894343 (Long-term follow-up of NCT05603312)	Parkinson's (GAD)	AAV (Not specified)	GAD	1 and 2	14	STN administration with sham surgery (Bilateral)	Ongoing study in PD patients: Assesses long-term follow-up safety study NCT05603312 (54 months for participants who received AAV-GAD and approximately 60 months for participants who received sham surgery)

Gene therapy approaches in AD

AD is a progressive, neurodegenerative disorder that impacts over 6.9 million Americans aged 65 and above [5]. AD is pathologically characterized by the extracellular deposition of Aβ plaques and intraneuronal accumulation of tau protein as NFTs. Available Food and Drug Administration (FDA)-approved medications, including acetylcholinesterase inhibitors such as donepezil, galantamine, and rivastigmine, offer marginal and short-term benefits, primarily addressing symptoms like cognitive impairment, mood enhancement (particularly apathy) and social interaction [30]. Recently approved disease-modifying therapies for AD, such as Aduhelm® (aducanumab-avwa), Leqembi® (lecanemab-irmb), and Kisunla® (donanemab-azbt), target Aβ plaques through distinct mechanisms and represent significant advancements in treatment [3]. These are humanized immunoglobulin gamma 1 (IgG_1_) monoclonal antibodies. Aducanumab-avwa binds to aggregated soluble forms (oligomers and protofibrils) and insoluble forms (fibrils) of Aβ, reducing Aβ plaques in the brain. Lecanemab-irmb targets aggregated soluble forms ) and insoluble forms of Aβ, preferentially binding to toxic protofibrils and preventing Aβ deposition before plaques develop while also removing existing plaques. Donanemab-azbt is directed against insoluble N-truncated pyroglutamate Aβ and helps remove Aβ plaques, slowing AD progression. However, Biogen has discontinued aducanumab due to reprioritizing resources allocated to aducanumab-avwa to advance lecanemab-irmb and to develop new treatment modalities [31]. The challenges related to ARIA, minimal clinical improvement, and limited efficacy in specific patient populations highlight the need for further advancements in AD treatment [3,31-34].

Given these limitations, gene therapy has emerged as a promising avenue for developing disease-modifying therapies for AD [3]. Gene therapy aims to enhance neuroprotection and reduce pathological Aβ aggregation, plaques, and NFTs by delivering therapeutic genes through viral vectors, such as AAV, to attenuate neuroinflammation and modulate neuronal function [2,3]. Preclinical investigations of nerve growth factor (NGF), brain-derived neurotrophic factor (BDNF), apolipoprotein E2 (APOE2), and human telomerase reverse transcriptase (hTERT) as potential therapeutic genes have demonstrated encouraging results in animal models [3]. These studies represent a significant step toward developing disease-modifying therapies that could potentially halt or even reverse the progression of AD, offering hope for a future where this devastating condition is no longer a death sentence.

As of June 2024, multiple gene therapy approaches are under investigation in clinical trials for AD. These trials target key molecular players implicated in AD pathogenesis, such as APOE2, BDNF, and hTERT (NCT03634007, NCT05400330, NCT05040217, and NCT04133454).

APOE2 Gene Therapy

The APOE gene, with its three isoforms (APOE2, APOE3, APOE4), plays a significant role in AD risk [35-38]. The APOE4 gene encodes for APOE4 protein, which is pathologically implicated in increasing AD risk and impaired lipid metabolism [35-38]. The isoforms are markedly variable in the degree to which they facilitate Aβ plaque and tau hyperphosphorylation [35,37,38]. Individuals who are homozygous for the APOE4 gene are at significantly increased risk of developing AD [37,39,40]. Clinical trials in humans were initiated following promising preclinical studies, which showed that AAV-APOE2 gene therapy reduced Aβ burden and safely achieved widespread distribution in the CNS [16,41]. A recent study in an AD mouse model suggested that APOE2 gene therapy reduced Aβ deposition, improved neuroinflammation markers, and prevented neurodegenerative synaptic loss [35].

Promising preclinical data in animal models have led to clinical trials investigating LX1001 gene therapy, designed to express the APOE2 protein in the CNS of individuals with AD. The ongoing phase 1/2 clinical trial of LX1001 consists of three dose-ascending cohorts. Initial data from the low-dose cohort demonstrated the detection of APOE2 protein in the CSF of all participants at three months post-treatment (Press release: LEXEO_LX1001_Biomarker_Data_Release_3.01_vF.pdf). Furthermore, in the two participants followed for one year, there were notable reductions in total tau and phosphorylated tau, key biomarkers implicated in AD progression. Notably, no serious adverse events were reported in either the low- or mid-dose cohorts, supporting the safety and tolerability of LX1001. 

These promising preliminary findings have led to the0 FDA granting LX1001 fast-track designation, underscoring its potential as a therapeutic intervention for AD. This designation will expedite the development and review process, enabling accelerated clinical trials and potentially faster access for patients.

Lexeo is also pursuing two additional clinical trials with their gene therapy candidates. One trial with LX1021 focuses on expressing the protective Christchurch-modified APOE2 protein in homozygous APOE4 carriers (NCT05400330). The other trial with LX1020 combines APOE2 expression with microRNA-mediated suppression of APOE4 expression (NCT04133454), potentially offering a dual therapeutic approach.

BDNF Gene Therapy

BDNF, a neuroprotective protein reduced in AD, has shown therapeutic potential in preclinical studies [42]. AAV-mediated BDNF delivery alleviated cognitive deficits, enhanced neuronal plasticity, and attenuated neuronal loss in transgenic AD models in aged rats [42]. In nonhuman primates, lentivirus-mediated BDNF delivery has shown promise in reversing neuronal atrophy and ameliorating age-related cognitive impairment [43]. Additionally, anterograde trafficking and MRI-guided infusion were found to be safe and accurate utilizing the AAV2 serotype [44]. These encouraging results have led the clinical trials investigating the efficacy and safety of BDNF gene therapy in AD patients.

An ongoing open-label phase 1 trial (NCT05040217) is investigating the delivery of AAV2-BDNF to the entorhinal cortex and hippocampus. The trial aims to assess safety, tolerability, and potential efficacy in early AD or MCI patients using a single-dose MR-guided stereotactic surgical injection. AAV2 is a safe and efficient vector for gene delivery to the CNS. The trial's primary endpoints include safety assessments and changes in memory function (assessed by the Ray Auditory Verbal Learning Task and Benson Complex Figure Draw and Memory test). Secondary endpoints encompass additional memory tests and changes in brain imaging. In addition, changes in biomarkers of disease progression, including Aß, tau, and neurofilament in the CSF, will be evaluated.

*hTERT Gene Therapy* 

Preclinical rodent studies utilizing an AAV9-mediated TERT gene therapy approach (AAV9-TERT) have demonstrated the potential of TERT, an enzyme vital for telomere maintenance, in mitigating neurodegeneration and age-related pathologies, including cognitive decline [44,45]. Furthermore, the approach enhanced neurogenesis and decreased glial fibrillary acidic protein (GFAP) expression, suggesting a decline in senescence and neuroinflammation [46].

Libella Gene Therapeutics sponsored a phase 1 clinical trial (NCT04133454) investigating the safety and tolerability of AAV-hTERT gene therapy in patients with neurodegenerative diseases, such as AD. This trial involved both IV and IT administration of AAV-hTERT in individuals aged 45 and older with early signs of dementia or AD diagnosis. The primary endpoints of the trial focused on the safety and tolerability of AAV-hTERT, while secondary endpoints assessed hTERT expression, telomerase activity, telomere length, and various measures of CNS changes and neurocognitive function, such as positron emission tomography (PET) imaging for amyloid, Trail Making Test (TMT) and the University of Pennsylvania Smell Identification Test ((NCT04133454). Although the trial's estimated completion date was January 2021, the results have not been made public.

In conclusion, while early clinical trial phases targeting APOE and BDNF have shown promising results, paving the way for potential disease-modifying treatments, the landscape of gene therapy in AD research is complex and evolving. Previous trials, such as the one involving CERE-110 (AAV-NGF), have highlighted the challenges in translating preclinical promise into clinical success. Despite initial safety and tolerability, CERE-110 failed to demonstrate a therapeutic effect in AD patients [47-51]. The ongoing AAV gene therapies (APOE2, BDNF, hTERT) trials represent significant advancements in the field. LX1001 targets high-risk individuals carrying the APOE4 allele and aims to prevent AD onset. Meanwhile, the AAV2-BDNF trial seeks to address both prevention and potential reversal of neurodegeneration by enhancing BDNF levels. These trials are currently in phase 1, and their outcomes will be critical in shaping the future direction of gene therapy research in AD. The development of safe and effective gene therapies remains challenging. Still, the potential to revolutionize the disease-modifying approach for AD offers new hope for millions affected by this heterogeneous disease. As research progresses, it is crucial to maintain a balanced perspective, acknowledging the promising early results and the lessons learned from previous trials.

Gene therapy approaches in PD

PD is the most prevalent movement disorder, affecting one million individuals in the US and over six million individuals worldwide [10]. The disease is primarily characterized by the progressive loss of dopamine-producing nerve cells in the substantia nigra pars compacta (SNpc) [2]. This neuronal loss is primarily caused by the accumulation and aggregation of misfolded α-Synuclein proteins, called Lewy bodies. Initially, motor function is impacted, leading to hallmark symptoms like bradykinesia, tremors, rigidity, and balance problems. However, as the disease progresses, the pathology extends to other brain areas, causing nonmotor symptoms such as cognitive decline, depression, anxiety, and hallucinations [9]. The exact causes of PD remain unclear, but it involves a combination of genetic predisposition, environmental factors, and aging processes. These factors contribute to cellular dysfunctions, including neuroinflammation, dysregulated cellular trafficking and mitochondrial function, oxidative stress, and endoplasmic reticulum stress [3,6,10,52].

Current therapeutic strategies for PD provide symptomatic relief but do not address the underlying neurodegenerative process. These treatments either supplement dopamine with drugs like levodopa/carbidopa or prevent its degradation using medications such as monoamine oxidase-B (MAO-B) inhibitors and catechol-O-methyltransferase (COMT) inhibitors [10]. While the drugs are initially effective, they often lose efficacy with time and further can cause debilitating side effects such as dyskinesia. Dyskinesia can be treated with deep brain stimulation (DBS), which has been shown to reduce symptoms and improve the quality of life in patients with PD. The limitations of current treatments, coupled with the heterogeneity of PD and its alarming global prevalence, underscore the urgent need for disease-modifying therapies that can alter the course of the disease.

Given the complex heterogeneity of PD and the limitations of current treatments, ongoing research into gene therapies aims to target the underlying causes of neurodegeneration, potentially slowing or even halting disease progression. These therapies restore dopaminergic function and neuroprotection, potentially slowing or halting disease progression. Several ongoing clinical trials are exploring gene therapy approaches, including those involving glial cell line-derived neurotrophic factor (GDNF), AADC, and glutamic acid decarboxylase (GAD) [10].

GDNF Gene Therapy

GDNF plays a crucial role in supporting the function and survival of dopaminergic neurons in the brain, which are significantly affected by PD. GDNF has emerged as a potential therapeutics using a gene therapy approach for PD [53-56].

Preclinical studies have demonstrated the promising impact of GDNF gene therapy; showcasing increased dopamine levels, improved motor and other behavioral symptoms, and protection and regeneration of these neurons [10,57-62]. These encouraging results from preclinical studies have paved the way for clinical trials investigating the safety and efficacy of GDNF gene therapy in humans.

GDNF gene therapy in human trials for PD began in 2013 with a phase 1 clinical trial (NCT01621581) [20]. This trial involved injecting AAV2-GDNF into the putamen of 13 participants using MRI-guided convection-enhanced delivery (CED). Participants received different doses of AAV2-GDNF [10]. While the procedure was safe and well-tolerated, PET scans indicated increased uptake of [^18^F]-FDOPA after six and 18 months. This indicated enhanced dopaminergic function at injection sites. Patients with shorter disease duration showed improvements earlier. After 18 months, no significant improvement in motor scores of PD or medication dosage was noted.

A recent conference presented findings from an open-label safety study (NCT04167540) that investigated the safety and potential clinical effects of AAV2-GDNF in individuals with recent or long-standing PD diagnoses [21]. The study included 11 participants, five in the mild and six in the moderate cohorts. Ten of the 11 completed the 18-month post-treatment assessment, and the mean putaminal coverage was 63% with a 2% standard error. Most of the treatment-emergent adverse events in all the participants were transient and perioperative. The study reported five serious adverse events, none of which were related to AAV2-GDNF gene therapy. In the mild cohort, scores in MDS-UPDRS, motor diary, unified dyskinesia rating scale (UDysRS), and levodopa equivalent daily dose (LEDD) remained stable. Interestingly, one participant in the mild cohort was found to have a heterozygous tyrosine hydroxylase mutation of unknown significance. The moderate cohort showed improvements from baseline at 18 months post-treatment in mean MDS-UPDRS Part III OFF scores, motor diary OFF time, UDysRS, and LEDD. To validate these encouraging findings, a phase 2 randomized controlled study is planned.

AADC Gene Therapy 

In PD patients, AADC activity is reduced, along with the loss of dopaminergic neurons. This reduced AADC activity has been linked to reduced conversion of L-DOPA to dopamine [63]. Therefore, restoring AADC levels in the brain emerges as a potential strategy for the management of PD. 

In preclinical animal models of Parkinsonian, AADC gene therapy using AAV2 has shown promising results in using neurotoxin-induced lesions [64,65]. Several studies in preclinical models have also co-expressed the genes for tyrosine hydroxylase (TH) and GTP cyclohydrolase I (GCH) along with AADC using AAV [63]. In PD patients, a reduction in the levels of TH, AADC, and tetrahydrobiopterin (BH4) levels has been shown in the striatum. GCH is a rate-limiting enzyme for BH4 biosynthesis, and BH4 is a cofactor of TH. Co-transduction of these genes, along with AADC, resulted in increased production of BH4 and dopamine and improved rotational behavior. After intrastriatal injection, these behavioral improvements persisted for at least 12 months (long-term). Extension of the observations led to the co-expression of TH, AADC, and GCH in the nonhuman primate model of PD [66,67]. The effective introduction of dopamine-synthesizing enzymes into the primate striatum restored motor function, and these behavioral recoveries persisted for several months [68]. In addition, AADC activity was persistent as measured with 6-[^18^F]fluoro-meta-tyrosine (FMT), a tracer for AADC [65]. These preclinical animal study findings led to the evaluation of AADC gene therapy in PD patients.

The AAV2-AADC (VY-AADC02) therapy is undergoing a phase 2 clinical trial (RESTORE-1; NCT03562494). In PD patients, the safety and tolerability of AAV2-hAADC have been evaluated in phase 1 trials, along with pharmacodynamics and preliminary efficacy [22,68-70]. These trials were conducted in the US and Japan, with the therapy being infused into the putamen of PD patients. At baseline and at six months post-infusion, various metrics were assessed, including UPDRS, motor state diaries, and PET with FMT. Six months post-surgery, in the OFF-medication state, there was an observed average improvement of 46% in motor functions, as indicated by UPDRS scores, with no significant changes in the short-duration response to levodopa [70]. An increase of 56% in FMT activity as measured by PET that persisted up to 96 weeks. 

In moderately advanced PD patients, an AAV2-hAADC bilateral intraputaminal infusion has been shown to improve mean scores on the UPDRS by about 30% in both the ON and OFF states in a phase 1 trial [68]. The study divided the 10 patients into two cohorts (low-dose and high-dose groups) of five each. Improvements were observed in both total and motor rating scales for both groups. Additionally, motor diaries reflected an increase in ON-time and a decrease in OFF-time, without increasing "on" time dyskinesia. In both cohorts at six months, PET scans using the AADC tracer FMT demonstrated a marked increase in putaminal uptake.

In a phase 1 study (NCT00229736), 10 moderately advanced PD patients who received a bilateral putaminal infusion of AAV2-hAADC (either a low or a high dose) were evaluated for the AADC expression using PET imaging with FMT. The study was a long-term follow-up study. In the first 12 months, an elevated PET signal was observed. In both low and high-dosage groups, the signal persisted over four years. Elevated expression revealed bilateral intraputaminal infusions of either a low or a high dose of the AAV [69]. In the first 12 months, in all patients, the UPDRS score OFF-medication improved, but in subsequent years, a slow deterioration was observed. Overall, over four years a stable transgene expression post-infusion was noted along with continued safety. The study highlighted the need for a controlled efficacy trial and further evaluation using a higher vector dose. 

In a clinical trial (NCT01973543) with 15 participants who had moderately advanced PD and medically refractory motor fluctuations, VY-AADC01 was administered bilaterally along with gadoteridol to the putamen, guided by intraoperative MRI [22]. AADC enzyme activity was measured at the baseline and six months post-procedure. The results indicated improved dose-dependent coverage of the putamen, increased AADC activity, and a reduction in the use of antiparkinsonian medication at six months. Furthermore, dose-dependent improvements in clinical outcomes were observed at 12 months, including increases in patient-reported ON-time without troublesome dyskinesia and improved quality of life.

Overall, few adverse effects (deep brain thrombosis, pulmonary embolism, atrial fibrillation, and intracranial hemorrhages) were noted in these clinical trials, and they were attributed to the surgical procedure [22,69,70]. The adverse effects were not related to gene transduction. A long-term observational extension study is ongoing to evaluate the safety of VY-AADC01 in subjects who have completed prior VY-AADC01 studies. The evaluation period extends to up to eight years following the administration of VY-AADC01 (NCT03733496).

An additional completed clinical trial (NCT03065192) evaluated VY-AADC01 in patients with advanced PD through MRI-guided convective infusion using a posterior trajectory into the putamen. Notably, this trial achieved greater than 50% total putaminal coverage, with a mean of 76% coverage of the post-commissural putamen. Refinements to the surgical procedure led to reduced infusion durations, shorter overall procedure times, and increased AADC enzymatic activity. Importantly, patients experienced a reduction in daily antiparkinsonian medication requirements at both six and 12 months post-VY-AADC01 administration. The findings from this trial (NCT03065192) directly informed the design of the subsequent RESTORE-1 trial (NCT03562494), refining surgical and infusion techniques. 

A multicenter-controlled phase 2 randomized trial was initiated to investigate the administration of VY-AADC02 (NBIb-1817)(NCT03562494; RESTORE-1). The primary outcome measure was to assess participants with treatment-emergent adverse events as a measurement of the primary outcome. However, the trial was placed on clinical hold due to T2 MRI abnormalities observed in patients, and the sponsor partnership ended in 2021 [70].

GAD Gene Therapy

The decrease in dopaminergic tone within the striatum of PD patients leads to heightened excitatory activity within the glutamatergic subthalamic nucleus (STN). The increased activity resulted in amplified depolarization, spike bursts, and subsequent amplified activation of the globus pallidus internus (GPi) and substantia nigra pars reticulata (SNr). This results in inhibitory effects on thalamocortical circuits, contributing to the motor symptoms observed in PD patients [10]. Furthermore, these regions exhibit reduced gamma-aminobutyric acid (GABA) concentrations, further exacerbating the motor symptoms [2]. The rate-limiting enzyme in forming GABA is GAD [27]. The motor features of PD could be improved by modulating STN activity. In this regard, the potential role of GAD gene therapy becomes particularly relevant, offering a promising therapeutic avenue for future research and treatment strategies for PD.

AAV-GAD gene therapy aims to increase GABA production within the STN. Preclinical studies in rat models demonstrated that transferring the GAD gene to excitatory STN neurons induced inhibitory responses and protected nigral dopamine neurons, effectively rescuing Parkinsonian behavior [10], suggesting the therapeutic potential of manipulating the balance between excitatory and inhibitory neurotransmission. Subsequent studies in hemiparkinsonian nonhuman primates showed that GAD gene therapy via AAV2 vector significantly improved bradykinesia, tremors, and gross motor skills [71]. In addition, it increased metabolism (glucose metabolism), and concentrated GAD expression in STN projections, all without adverse effects. The AAV-GAD treatment was safe and well-tolerated throughout the 55-week follow-up period in NHP models, with no adverse events reported. Notably, the treatment demonstrated stable and significant long-term improvements in motor function. This approach, targeting STN downregulation rather than dopamine restoration, broadened the scope of potential PD treatments. These promising preclinical and nonhuman primate results provided a strong rationale for initiating human clinical trials to investigate the safety and efficacy of AAV-GAD gene therapy in PD patients.

Phase I and II clinical trials (NCT00643890; NCT00195143) examined the effects of GAD delivered to the STN in PD patients. Compared to the placebo group, patients treated with AAV2-GAD gene therapy demonstrated significant improvements in motor function according to UPDRS motor scores 12 months post-infusion. They also experienced a reduction in the duration of levodopa-induced dyskinesia, a decrease in self-reported OFF time, an increase in self-reported ON time, and a reduction in thalamic metabolism [26-28]. The treatment was well-tolerated, with only minor side effects like nausea and headache reported [27]. These findings underscore GAD transduction's effectiveness in modulating STN overactivity and highlight the potential of restoring the GABAergic pathway to alleviate PD symptoms.

Ongoing clinical trials are evaluating the safety and efficacy of bilateral AAV-GAD administration to the STN in patients with PD. These include randomized, double-blinded, sham-controlled studies (NCT05603312) and a long-term follow-up safety study (NCT05894343). These investigations hold promise for expanding treatment options for PD and improving patient outcomes by offering a nondopaminergic therapeutic approach that could be combined with existing treatments for enhanced efficacy.

In conclusion, the development of gene therapies for PD is a rapidly evolving field with the potential to revolutionize treatment approaches. The promising results from preclinical and clinical trials of GDNF, AADC, and GAD gene therapies highlight the potential of these approaches to modify the course of the disease, potentially slowing or even halting its progression. While challenges remain in optimizing delivery methods, dosages, and long-term efficacy, the ongoing research and clinical trials offer hope for a future where PD can be effectively managed or even cured. The diversity of gene therapy targets also underscores the importance of personalized medicine, where treatment can be tailored to each patient's specific needs and genetic profile. As research advances, gene therapy promises to transform the lives of millions of people affected by PD worldwide.

Challenges and potential risks of gene therapy in AD and PD

Gene therapy offers a promising approach for treating neurodegenerative diseases like AD and PD, but several common challenges and potential risks need to be addressed for successful clinical translation.

Disease Heterogeneity and Clinical Trial Design

AD and PD are complex and heterogeneous diseases with varying underlying pathologies, necessitating personalized approaches, including developing multidrug strategies and identifying biomarkers for early detection. In the future, as more gene therapies receive FDA approval, it will be valuable to explore in the clinical trial the combination of different molecular therapies along with gene therapy targeting various molecular targets based on the patient's disease state and genetic profile. This approach may aim to achieve optimal disease modification and alleviate disease progression. For example, the ongoing clinical trial (NCT04133454) that combines APOE2 expression with microRNA-mediated suppression of APOE4 expression potentially exploring a dual therapeutic approach. Considering disease heterogeneity, it is pertinent to address patient selection criteria and enrollment issues and define the primary and secondary outcomes well. This will help ensure the relevance of the gene therapy and the generalizability of trial findings.

Blood-Brain Barrier (BBB) Penetration

The BBB poses a significant challenge in delivering therapeutic biotherapeutics, such as transgene of interest, using gene therapy for AD and PD to the brain. Efficiently crossing this barrier is crucial for successful gene delivery. To achieve this, ongoing research is focused on developing BBB-penetrating AAV capsids, exploring alternative delivery methods like nonviral vectors and focused ultrasound, and investigating strategies to open the BBB transiently [72,73].

Target Specificity and Transduction Efficiency

To treat neurological disorders, it is essential to precisely deliver the therapeutic gene of interest to specific brain regions or cell types with minimal adverse effects. Utilizing specific AAV serotypes, optimizing administration routes, and improving diagnostic tools can enhance target specificity and transduction efficiency [14-16,40]. As it has been reported that neutralizing antibodies (NAbs) against AAV vectors can deter therapeutic efficacy, especially during re-administration, strategies are needed to prevent Nab generation.

Long-Term Gene Expression and Safety

Considering the chronic progressive nature of neurodegenerative diseases, stable and long-lasting therapeutic transgene expression is essential for sustained therapeutic benefit [74]. Long-term gene expression would be critical in minimizing re-administration, thereby reducing the risk of triggering immune response or toxicity and increasing the cost associated with the therapeutic approach. Various research approaches are focused on engineering less immunogenic viral serotypes and incorporating inducible or regulatable gene expression systems [14].

Affordability and Accessibility

The high cost of gene therapy development and production raises concerns about affordability and accessibility for patients, especially those with limited financial resources [75,76]. Collaborative efforts between researchers, clinicians, and industry partners are needed to address this challenge and ensure equitable access to these potentially life-changing treatments.

*Utilization of Approved Blood-Based Biomarkers* 

Incorporating a comprehensive panel of emerging blood-based biomarkers throughout the clinical trials process would allow the determination of the interim effects on the key pathological process and biological pathways with enhanced safety monitoring. Further, longitudinal measurements of these biomarkers could also help assess the disease-modifying relevant impact of the gene therapies once received by AD and PD patients.

In summary, while gene therapy holds immense potential to revolutionize the treatment landscape for AD and PD, addressing these common challenges and potential risks is crucial for its successful clinical translation. Continued research and development efforts, along with collaborative initiatives, are necessary to overcome these obstacles and make gene therapy a viable and accessible treatment option for millions affected by these debilitating neurodegenerative diseases.

## Conclusions

Gene therapy, a disease-modifying approach utilized in clinical trials for AD and PD, holds immense promise for patients affected by these chronic neurodegenerative diseases. In the early phases of clinical trials evaluating safety and toxicity, promising results have been observed in several clinical trials. In the later stages, the efficacy of the gene therapy approach in alleviating the neuropathology and clinical outcomes underlying these diseases is under evaluation. Significant challenges remain in optimizing delivery methods, dosages, and long-term effectiveness, minimizing the serious adverse effects. Future research should focus on identifying the most suitable patient populations (genetic profile, family history, and other risk factors) utilizing biomarkers and imaging studies, developing more precise gene editing tools, and addressing safety concerns. Despite these challenges, the potential of gene therapy to modify the course of AD and PD brings hope to millions of patients worldwide.
